# Social and Poor-Law Problems

**Published:** 1908-07-25

**Authors:** 


### SOCIAL AND POOR-LAW PROBLEMS.

### THE BOARDING OUT OF POOR-LAW CHILDREN.

The annual report of the Local Government Board contains some interesting facts concerning the results of boarding out children in cottage homes. The senior lady inspector, Miss Mason, collected and tabulated the after-histories and occupations of more than 1,800 boys and girls who had been boarded out in different parts of the country, and the result was to prove that in the abodes of their foster-parents the children had found a real home, such as, in Miss Mason's opinion, "no institutions, however small and home-like," could equal. She adds: "Neither, as I have often shown, can any other system afford homes to which these children can return in after life, when out of place or on holidays." This is an important point, for the best institutions can make no provision for the children when they have ceased to be chargeable if they are in temporary need of a home, except by offering the workhouse. Any plan that will keep a Poor-law child from feeling that the Union is the only place he can fall back upon in emergencies is valuable, and this aspect of boarding out should not be forgotten. Another matter of importance is the more independent spirit which the children possess when they are brought up in the ordinary surroundings of a home of their own class, rather than in an institution where each must be more or less only one of a crowd. They are encouraged to save any little money they receive, and in many cases they have by the time they enter the world considerable sums to their credit in the savings bank. As a rule the bank-books are kept by the secretary of the local committee. Both Miss Mason and some of the other inspectors have most encouraging reports of the success of the Poor-law children. One boy who was learning engineering withdrew some of his savings to pay for evening classes at a technical school. Miss Walton Evans tells of one boy who had gained a scholarship of the annual value of £15 at an old-established grammar school, and several girls are competing for scholarships. This mingling on ordinary terms with other children—whether or not they gain scholarships—is a great help to the children's future self-respect and independence of character. But certainly boarding out places a great responsibility on the local committee, for though the Local Government Board inspectors may do their best, they cannot pay the frequent surprise visits which are necessary to discover if a child is well-cared for and happy. Carelessness or laxity on the part of a committee in a single case may so prejudice a board of guardians that they will give up boarding out altogether, on the ground that Poor-law money should be expended only under direct control. It is unfortunte that so many boards are—one can call it nothing else—so mean in regard to boarding out. In one case foster-parents were offered only 3s. 6d. a week for the maintenance of a child—a rate so unremunerative that no one would accept it. Yet these same children in institutions often cost as much as 10s. a week per head, and the guardians never grudge it! Another thing that weighs with boards is the cost of defraying the expenses of visiting guardians. This is just the sort of expenditure which, if the public hears of it, is used to make an outcry against guardians on the ground of extravagance, and "taking holidays at the expense of the ratepayers." Yet these visits of inspection are necessary, and they are usually expensive, for, as a rule, it is better for a boarded-out child—who has often undesirable friends—to be sent a considerable distance from the Union. It is true that these journeys are as a rule more of a fatigue than a pleasure, but the public does not know that. But such considerations should not weigh with those who believe that boarding out is the best way to bring up any of the children under their care. And when there is a careful choice of foster-parents there can be little doubt that this method, because it most nearly approaches to natural home conditions, and tends to rear the children as intelligent and independent citizens. And in this connection it is pleasant to quote Miss Mason's testimony to foster-parents as a whole. "No one," she says, "appreciates more than I do the good work done by many foster-parents; that the love and care given the children from the highest motives is such as no money can adequately pay for, and examples of honesty and industry are set that fill one with admiration."

